# Radiation Hardness of Silicon Carbide upon High-Temperature Electron and Proton Irradiation

**DOI:** 10.3390/ma14174976

**Published:** 2021-08-31

**Authors:** Alexander A. Lebedev, Vitali V. Kozlovski, Klavdia S. Davydovskaya, Mikhail E. Levinshtein

**Affiliations:** 1 Solid State Electronic Department, Ioffe Institute, Politekhnicheskaya Street 26, 194021 St. Petersburg, Russia; Davidovskaya.Klava@mail.ioffe.ru (K.S.D.); melev@nimis.ioffe.ru (M.E.L.); 2 Department of Experimental Physics, St. Petersburg State Polytechnic University, Polytekhnicheskaya 29, 195251 St. Petersburg, Russia; kozlovski@physics.spbstu.ru

**Keywords:** silicon carbide, radiation hardness, proton and electron irradiation, charge removal rate, compensation, irradiation temperature

## Abstract

The radiation hardness of silicon carbide with respect to electron and proton irradiation and its dependence on the irradiation temperature are analyzed. It is shown that the main mechanism of SiC compensation is the formation of deep acceptor levels. With increasing the irradiation temperature, the probability of the formation of these centers decreases, and they are partly annealed out. As a result, the carrier removal rate in SiC becomes ~6 orders of magnitude lower in the case of irradiation at 500 °C. Once again, this proves that silicon carbide is promising as a material for high-temperature electronics devices.

## 1. Introduction

One of the stimuli in development of the technology of wide-bandgap semiconductors and creating devices on their basis is the high presumed radiation hardness of these materials. Indeed, making higher binding energy of atoms in the lattice of a semiconductor requires a higher energy of particles needed to disintegrate this lattice. Studies carried out in the 1960s demonstrated that silicon carbide substantially surpasses silicon in the radiation hardness [1]. Later, with increasing structural perfection of SiC and decreasing level of background doping, the difference in radiation hardness between SiC and Si decreased. It is noteworthy that the decrease in radiation hardness with the increasing quality of material is also characteristic for other semiconductors. Various structural defects and uncontrollable impurities could serve as drains for radiation defects and, thereby, slow the degradation of material parameters.

However, statements started to appear in the literature suggesting that the radiation hardness of silicon carbide does not surpass, and is even inferior to that of silicon in certain conditions [2,3,4,5]. This conclusion seems to be surprising because the energy gap of 4H-SiC (3.2 eV) is nearly three times that of silicon. We found it interesting to consider the situation by using both our results and those of other researchers. Thus, the goal of the present study is to consider the issue of the radiation hardness of silicon carbide and compare it with the similar characteristic for Si.

Our work is focused on the results of high-temperature irradiation, due to the fact that a lot of works have been devoted to the study and analysis of the results of irradiation at room temperature. The great number of published studies are concerned with the radiation hardness of SiC MOSFETs against γirradiation [6,7,8,9,10,11,12]. The effect of room temperature electron irradiation on the properties of high-voltage 4H-SiC Schottky diodes also has been studied in many works [13,14,15,16,17,18,19,20]. The effect of room-temperature proton irradiation on the properties of 4H-SiC JBS has been extensively studied [21,22,23,24,25,26,27,28]. Consequently, in this work, we considered it expedient to focus on the results of our work in the field of high-temperature irradiation.

## 2. Mechanism of Radiation-Induced Degradation of SiC

The radiation-induced degradation of a semiconductor device is commonly understood as the deterioration of its performance under irradiation with high-energy particles. The higher the irradiation dose required for the degradation of a semiconductor, the more radiation-hard it is believed to be.

First, consider the possible mechanisms of the radiation-induced conductivity compensation [29,30].

Assuming, for example, that the main defects generated by fast electrons are vacancies in a SiC sublattice; if the formation of multivacancy complexes is considered unlikely, we have

(1) $$\frac{\mathrm{dV}}{\mathrm{dt}}=\eta_{\mathrm{FP}}\cdot G-\frac{V}{\tau }-\beta \cdot V\cdot N$$

Here V is the concentration of vacancies, G the flux of charged particles, η_FT_ the probability of vacancy formation by a single particle, τ the lifetime of a vacancy, determined by drains; β the probability of vacancy capture by a free (having no captured vacancy) atom of nitrogen impurity, and N the concentration of free nitrogen atoms. The initial conditions are t = 0, V = 0, N = N_0_.

The concentration of complexes of secondary defects N_c_ (vacancy and impurity atom) can be calculated by the formula

N_c_ = N_0_ − N (2)

where N_c_ being zero at the initial moment of time. The concentration of carriers, electrons (n), is the difference between the concentrations of impurities (shallow donors) and complexes (in the case of deep acceptors).

n = N – N_c_ = 2N − N_0_ (3)

Assuming that the lifetime of a vacancy, determined both by drains and by the impurity capture, substantially exceeds the irradiation time, then, the term V/τ in Equation (1) can be neglected.

A semiconductor can be compensated by two mechanisms. First, the radiation-induced defects create deep acceptor levels, to which electrons from shallow donor levels pass. In this case, no vacancy donor level complexes are formed.

Then, the concentration of vacancies linearly grows with increasing irradiation dose

V = η_FP_·G (4)

and the carrier concentration linearly falls:

N = N_0_ − η_FP_·G (5)

Thus, with this mechanism being operative, the carrier concentration will linearly decrease with increasing irradiation dose.

In the framework of the second mechanism, the radiation defect (vacancy) interacts with a shallow-impurity atom to give an electrically neutral (or acceptor) center. This occurs when the lifetime of a vacancy is substantially shorter than the irradiation duration, being determined by drains. In this case, the vacancy concentration can be considered stationary and be determined from Equation (1) as

V = η_FP_·G·τ (6)

In this case, the dependence of the carrier concentration on the irradiation dose is determined by the interaction of a vacancy with an impurity. Thus, the contribution of the secondary radiation defects, vacancy + impurity atom complexes, dominates. In this case, the carrier concentration is equal to the concentration of free impurity atoms (N), n = N.

The kinetics of N with second mechanism is described by the equation

(7) $$-\frac{\mathrm{dN}}{\mathrm{dt}}=\eta_{\mathrm{FP}}\cdot \beta \cdot G\cdot \tau \cdot N$$

possessing the following analytical solution

N = N_0_ exp(−η_FP_ β·τ·G·t) (8)

In this case, the concentration of the electrically active impurity will exponentially decrease with increasing irradiation dose.

**Figure 1:**
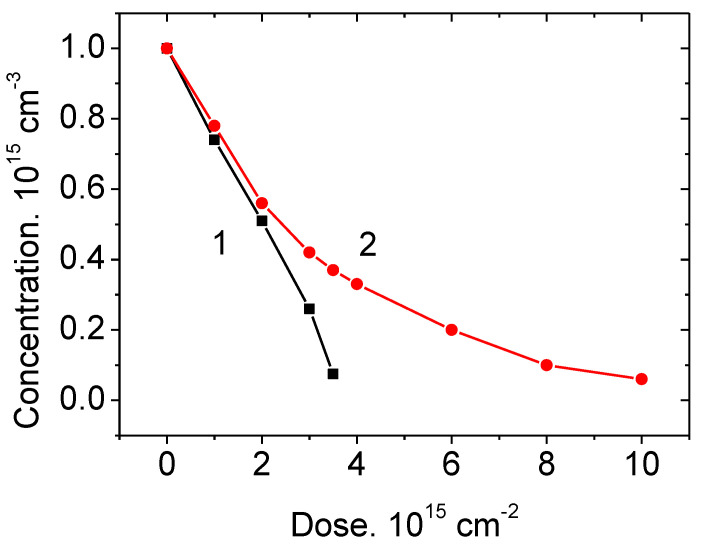
Conductivity compensation in (1) n-4H-SiC and (2) n-Si under irradiation with 0.9 MeV electrons. Points represent experimental data. The straight line 1 represents a calculation according to Equation (9) at a parameter η_FP_ of 0.25 cm^−1^. Curve 2 represents a calculation by Equations (5) and (8) at a factor (η_FP_ β τ) in the exponent equal to 1.2 × 10^−16^ cm^2^.

Figure 1 shows experimental data for N_d_–N_a_ = F(ΔD) for SiC and Si, where ΔD is the irradiation dose.

As can be seen from Figure 1, for silicon carbide, in the similarity to GaAs, the carrier concentration linearly decreases with increasing irradiation dose. This means that the first compensation mechanism is operative in SiC, this mechanism being associated with the formation of deep acceptor levels and transition to these levels of electrons from shallow donors. The linear dependence of the carrier concentration on the irradiation dose has also been observed in studies by other researchers, see, e.g., [31,32].

## 3. Experiments on Determining the Carrier Removal Rate

Frequently, the radiation hardness of a semiconductor is evaluated by the parameter “carrier removal rate” V_d_, defined by

(9) $$V_{d}=\frac{N_{0}-N_{1}}{\Delta D}$$

where N_0_ is the concentration N_a_–N_d_ in the epitaxial layer prior to irradiation; N_1_ the N_a_–N_d_ concentration in the epitaxial layer after the irradiation; and ΔD the irradiation dose.

The value of V_d_ for Schottky diodes (SBDs) and junction-barrier Schottky (JBS) diodes under irradiation with electrons and protons are listed in Table 1, which also presents the carrier removal rates for silicon under the same irradiation conditions. It can be seen that V_d_ for SiC is approximately twice as small as that for Si.

**Figure 2:**
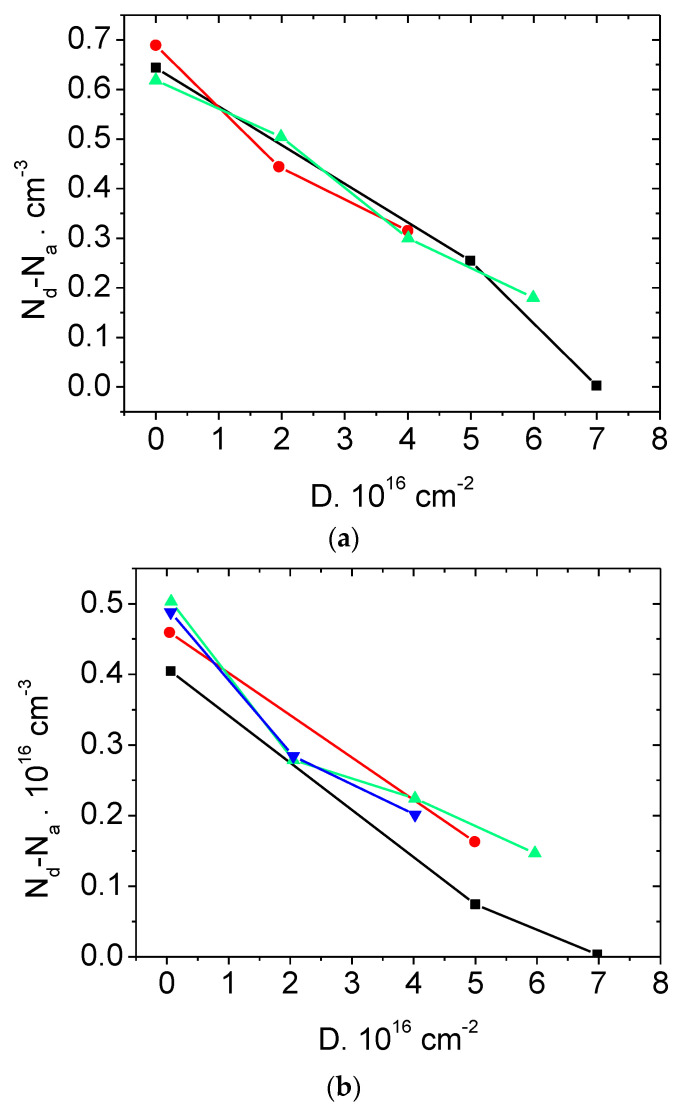
Dependence of the concentration N_d_–N_a_ in Schottky diodes (CREE) with the blocking voltage of (**a**) 600 V and (**b**) 1200 V on the electron irradiation dose at room temperature. Different symbols correspond to different diodes from the same batch.

**Figure 3:**
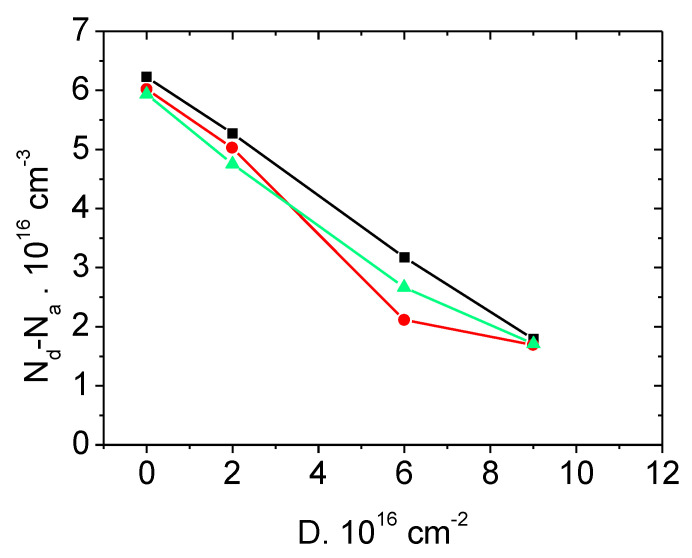
Dependence of the concentration N_d_–N_a_ in Schottky diodes (CREE) with the blocking voltage of 600 V. Irradiation with protons at room temperature. Different symbols correspond to different diodes from the same batch.

Figure 2 and Figure 3 present the dependences N_d_–N_a_ = F(ΔD) under irradiation with electrons and protons of SiC Schottky diodes manufactured by CREE company. It can be seen that this dependence is linear, which confirms the conclusion made in Section 2 about the mechanism of the radiation compensation of SiC via formation of deep acceptor levels.

## 4. Degradation of SiC Performance under the Action of Radiation

When a charged particle is decelerated in the semiconductor matrix, the released energy can shift the lattice atoms away from the equilibrium position. This yields the so-called primary radiation defects (Frenkel pairs), vacancies in the lattice and interstitial atoms. Most of formed defects recombine, and the rest of them create levels (deep centers) in the band energy gap of a semiconductor. Also possible is the interaction of primary defects with each other and with impurity atoms to give secondary radiation defects. As a rule, the secondary radiation defects are formed upon an increase in temperature, which is accompanied by the annealing out of the remaining primary defects.

As the irradiation dose increases, radiation defects gradually accumulate, which causes degradation of a semiconductor device, for example, see [36,37,38]. In SiC pn structures, as well as in pn structures based on other semiconductor materials, irradiation leads to the following effects.

1. The free carrier concentration decreases and, accordingly, the ohmic resistance of the base region grows. This is due to the formation of compensating radiation defects to which free carriers go.
2. The carrier lifetime and their diffusion length become shorter. This is due to the increase in the concentration of recombination centers in the semiconductor.
3. The leakage currents under a reverse bias increase. This may be due to the formation of defect clusters shunting the pn junction.

**Figure 4:**
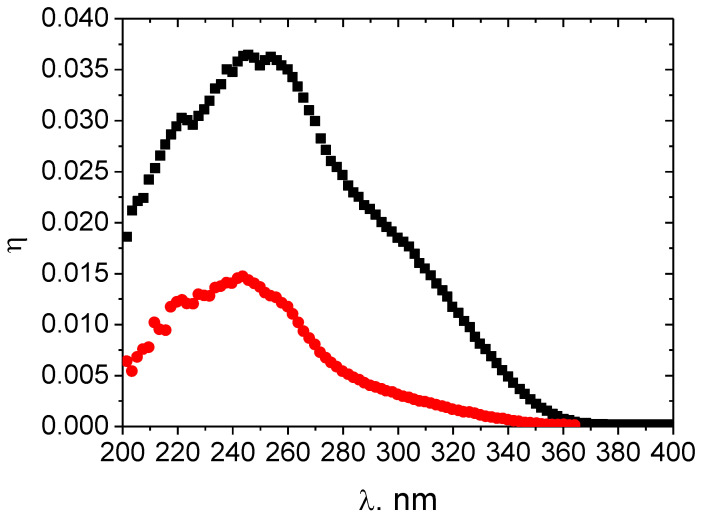
Spectral dependences of the quantum efficiency of a 4H-SiC photodetector with Schottky barriers: (black) initial sample and (red) sample irradiated with 167 MeV Xe ions at a fluence of 6 × 10^9^ cm^−2^. Room temperature.

Figure 4 shows dependences of the quantum efficiency of a SiC UV photodetector before and after the irradiation with heavy ions. The decrease in the quantum efficiency is due to that in the carrier diffusion length.

**Figure 5:**
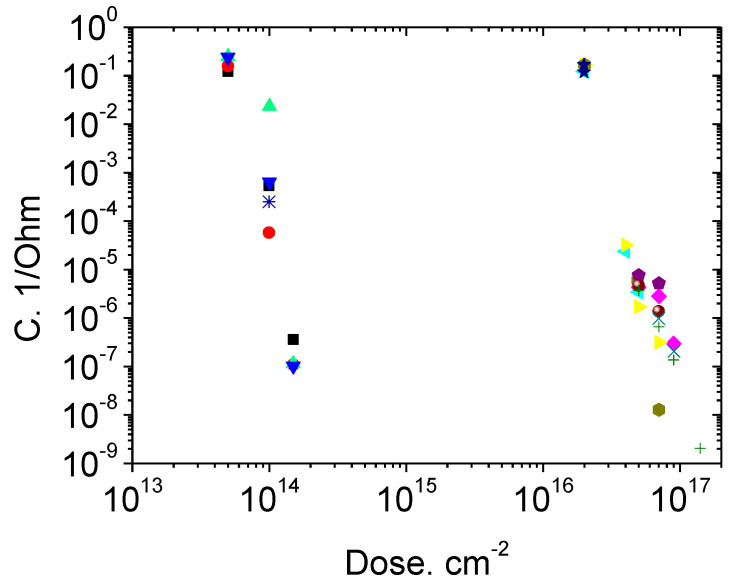
Base conductivity of a Schottky diode (600 V) after (1) protons and (2) electron irradiation. Different symbols correspond to different diodes from the same batch.

Figure 5 shows how the conductivity of Schottky diode bases decreases upon irradiation with protons and electrons. The formation of compensating acceptor levels leads to a 6–7 orders of magnitude decrease in the carrier concentration in the base region. In this case, the carrier mobility decreases only slightly.

**Figure 6:**
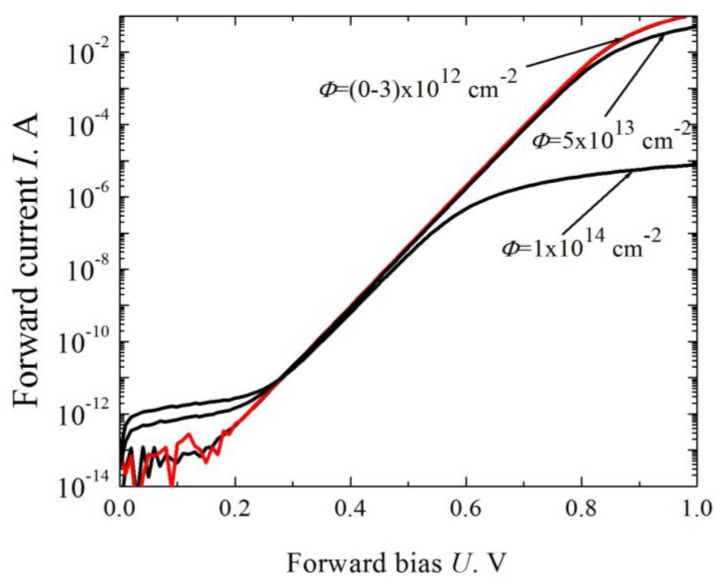
Forward current-voltage characteristic of a Schottky diode (1200 V class) at various doses of irradiation with 15 MeV protons [36].

Figure 6 shows current-voltage characteristics of a Schottky diode with breakdown voltage of 1200 V after the proton irradiation. The irradiation affects only slightly the voltage dependence of the forward current in the exponential area of the current-voltage characteristic. The irradiation in the pre-exponential (currents of 10^−12^–10^−14^ A) and post-exponential (high currents) areas drastically affects the current-voltage characteristic. At high currents, the base resistance increases due to the decrease in the free-carrier concentration. Leakage currents grow at low currents.

The reverse current-voltage characteristics of Schottky diodes before and after the proton irradiation were investigated in [33]. It was shown that the leakage currents decrease at low reverse voltages, which is apparently due to the total increase in the resistance of the structure. At high reverse voltages, the reverse currents do increase, which can be attributed to the appearance in the space-charge layer of deep centers (radiation defects) via which carriers are generated.

**Figure 7:**
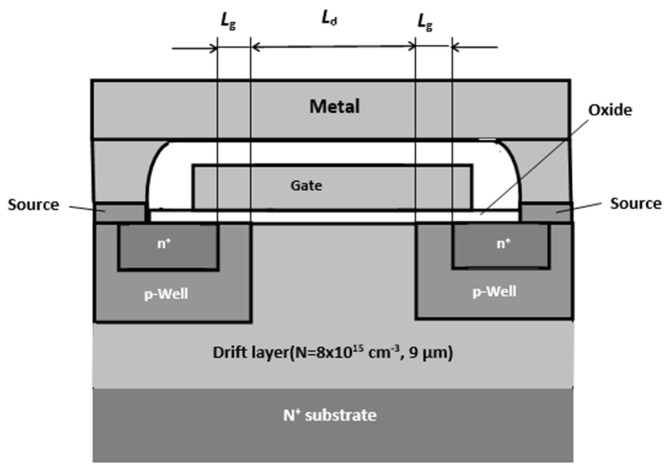
Cross-section of an elementary cell of a 4H-SiC MOSFET. The gate length Lg is 0.5 μm, the oxide thickness d is 60 nm, and the drift (blocking) layer thickness Hd is 9 μm [37].

**Figure 8:**
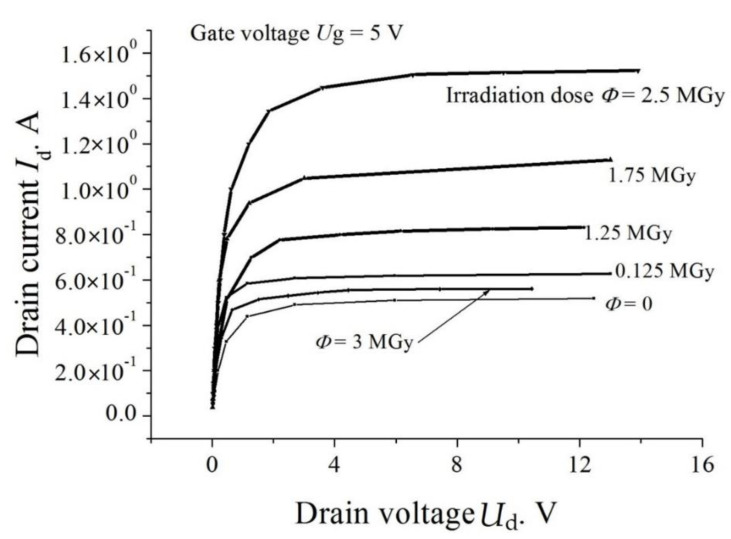
Output characteristics I_d_(V_d_) of a SiC MOSFETs (1.2 kV class) under study at various irradiation doses. The gate voltage V_g_ = 5 V [37].

Somewhat more complicated is the result of irradiation of SiC MOSFETs (a typical structure of such a device is shown in Figure 7). First, as in the case of a pn structure and Schottky diode, the free-carrier concentration decreases and the resistance of the drift region grows. Second, the devices have a subgate insulator layer (SiO_2_) in which the charge state of traps changes under irradiation. This may lead to an increase in the output current of a transistor at small irradiation doses. Both of these effects are well represented in the current-voltage characteristics presented in Figure 8.

## 5. Comparison of the Radiation Hardnesses of Si and SiC

Since silicon carbide is often viewed as a possible replacement for silicon in power devices, it is interesting and useful to compare the radiation hardness of the two materials. In our opinion, two approaches to such a comparison are possible.

First, two SiC- and Si-based diodes with the same breakdown voltage can be compared

U_brSi_ = U_brSiC_ => (E_crSi_·W_Si_)/2 = (E_crSiC_·W_SiC_)/2 => W_cr_ = W_Si_·E_crSiC_/E_crSi_ (10)

Here, U_br_ is the breakdown voltage, E_cr_ the critical electric field, and W the space-charge layer thickness at U_br_.

Because the critical field in silicon carbide exceeds by an order of magnitude that in silicon, E_crSiC_/E_crSi_ ≈ 10, we obtain, with consideration for the fact that W ≈ $\surd N_{d}-N_{a}$, N_(d−a)Si_ = 100 N_(d−a)SiC_, where N_d−a_ is the concentration of the uncompensated impurity in the base.

Thus, at the same breakdown voltage, the SiC diode is doped to a level exceeding by two orders of magnitude that for the Si diode. Consequently, even at equal values of V_d_, the compensation of SiC diodes requires a 100 times higher irradiation doses, compared with Si diodes.

Second, the radiation hardness of SiC and Si diodes with the same base thickness can be compared [39]. This is important for fabrication of charged-particle detectors, in which the applied reverse voltage is limited and the maximum thickness of the space-charge layer should be obtained. In this case, the carrier-removal rates are directly compared.

It can be seen in Table 1 that the value of V_d_ for SiC is only two times smaller than for Si. Because the energy gap E_g_ of SiC is nearly three times that for silicon, a question arises why the difference between the values of V_d_ for these two materials is so insignificant.

Table 2 presents the results of an analysis of the annealing-out of radiation defects in 4H and 6H silicon carbide irradiated with various kinds of ions. It can be seen that there are two characteristic temperature ranges in which this annealing occurs: 200–800 °C and ≥1200 °C. Such a situation is also characteristic of other semiconducting materials. In the first stage of annealing, most of the primary radiation defects recombine, with the remaining forming substantially more temperature-resistant complexes, which are annealed out at significantly higher temperatures. However, the position of these annealing stages along the temperature scale depends on the properties of a semiconductor, including its energy gap.

**Figure 9:**
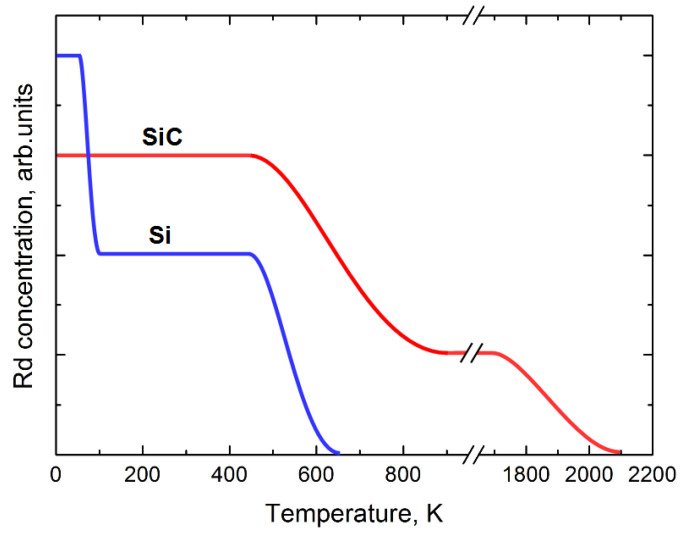
Schematic comparison of how the radiation defects are annealed-out in SiC and Si [38].

Figure 9 schematically demonstrates how the concentration of radiation defects (R_d_) in silicon and silicon carbide varies with temperature.

The figure shows that, at room temperature, these two materials are in different physical states with respect to the annealing-out of radiation defects. For silicon, the primary annealing stage has already been completed, whereas for SiC it has not yet begun. Thus, even if the concentration of introduced radiation defects was lower immediately after the irradiation (the irradiation temperature is conditionally 0 K), the concentration of defects in silicon when heated to room temperature became lower than that in SiC.

This can explain such a small difference between V_d_ in SiC and Si and gives impetus to a desire to verify this assumption and irradiate silicon carbide at elevated temperatures.

## 6. Irradiation of SiC at Elevated Temperatures

Previous experiments with III–V materials have demonstrated that the irradiation temperature can cardinally change the radiation hardness of materials and devices [46]. We examined, for the first time, the influence exerted by the electron irradiation temperature on high-power (blocking voltage 1700 V, working current 10 A) 4H-SiC Schottky diodes within the range 23–500 °C [47,48,49].

To perform high-temperature irradiations, we designed and constructed a special target chamber. This chamber enabled us to work with irradiations in air at temperatures ranging from room temperature to 600 °C. The accuracy of maintaining the sample temperature during irradiation was ±5 °C. The heating rate was maintained at 0.5 deg/s and the cooling rate was about 0.25 deg/s.

**Figure 10:**
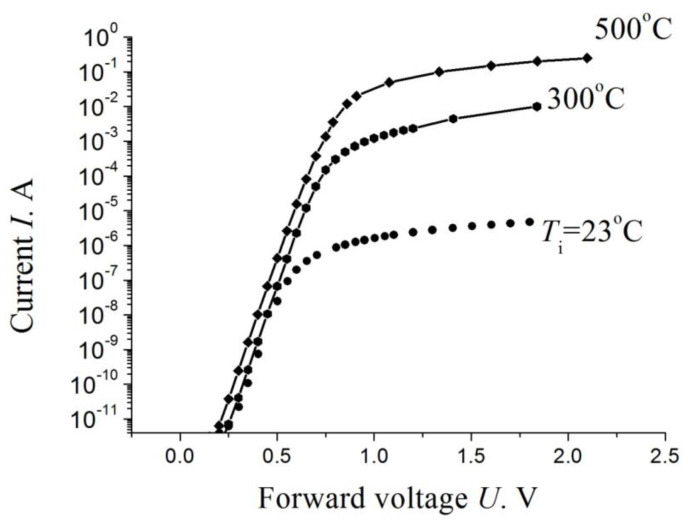
Forward current–voltage characteristics of diodes upon their irradiation with 0.9 eV electrons at three different irradiation temperatures *T*_i_. the dose *Φ* ≈ 6 × 10^16^ cm^−2^. The inset shows how the base resistivity ρ depends on the inverse irradiation temperature [47].

It was found that, at comparatively small values of *Φ* ≈ 10^16^ cm^−2^, raising the irradiation temperature from room temperature to 300 °C affects, comparatively slightly, the electron removal rate. With increasing dose, the difference between the base resistivities upon irradiation at room and elevated temperatures monotonically grows and exceeds three orders of magnitude at *Φ* ≈ 6 × 10^16^ cm^−2^ (Figure 10).

We also examined the effect of a high-temperature irradiation with 15 MeV protons on parameters of high-voltage 4H-SiC Schottky diodes at doses in the range from 7 × 10^13^ to 2 × 10^14^ cm^−2^.

**Figure 11:**
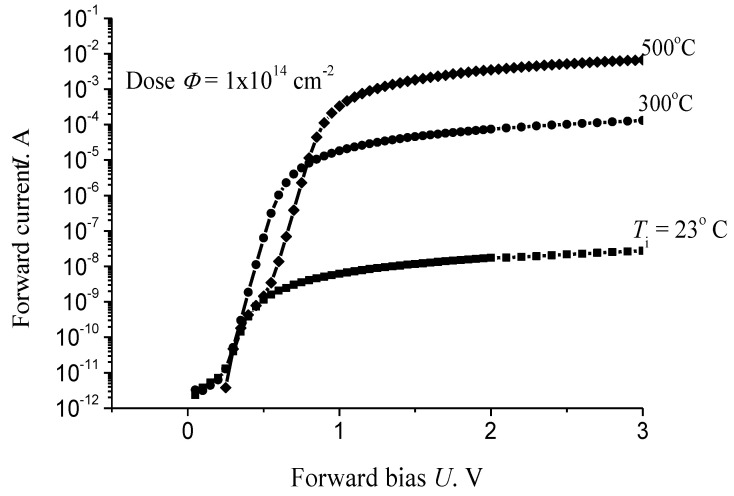
Forward current–voltage characteristics of the diodes upon their irradiation with 15 MeV protons at three different irradiation temperatures *T*_i_, the dose *Φ* = 1 × 10^14^ cm^−2^ [48].

After the irradiation with a dose of 10^14^ cm^−2^ at room temperature, the forward current at a forward voltage U = 2 V decreases by ~10 orders of magnitude (Figure 11). In this case, the cutoff voltage U_c_, equal to ~0.6 V in unirradiated devices, decreases to U_c_ ≈ 0.35 V. By contrast, irradiation with the same dose at a temperature of 500 °C results in that U_c_ increases to U_c_ ≈ 0.8 V. At the same reference forward voltage U = 2 V, the decrease in current, as compared with the value for unirradiated devices, was ~4 orders of magnitude. In the whole range of doses and irradiation temperatures under study, the forward current-voltage characteristic of the diodes is linear at U > U_c_ up to U ≥ 2 V. In unirradiated diodes, the forward current I at the reference forward voltage U = 2 V is I ≈ 12 A (see details, datasheet, quote on part number: CPW3-1700-S010B-WP).

The resulting set of experimental data indicates an increase in the radiation resistance of diodes with an increase in the temperature of irradiation. The physical reason for this temperature dependence is a decrease in the stationary concentration of radiation defects (RD), which are responsible for compensation of the base conductivity of the Schottky diodes under study, with an increase in the irradiation temperature.

As is known, the main RDs that create deep acceptor levels in n-SiC are mainly carbon vacancies [37,50]. The rate of generation of primary RDs (which are vacancies and interstitial atoms in both silicon carbide sublattices) in the temperature range under study is practically independent of the irradiation temperature [51,52].

However, the further fate of the generated vacancies (secondary defect formation) can significantly depend on temperature. As the temperature rises, the vacancy mobility increases and the recombination radius with a genetically related interstitial atom increases. Therefore, the fraction of vacancies that have escaped recombination and created deep acceptor levels is greatly reduced. According to our picture of irradiation, this proportion is about 25%, then at 300–400 °C it decreases by a factor of 2–3.

In principle, a second possible reason cannot be ruled out, which is a change in the spectrum of secondary radiation defects created during hot irradiation. A change in the X-ray diffraction spectrum was previously observed under hot electron irradiation of silicon and A_3_B_5_ materials [47,52].

Thus, we can conclude that the previously made assumption that the radiation hardness of silicon carbide will grow with an increase in the irradiation temperature is valid. In our opinion, this is an important conclusion because SiC is considered to be a promising material, especially for development of high-power and high-voltage devices.

## 7. Conclusions

- Silicon carbide of *n*-type is compensated under irradiation due to the transition of carriers to the acceptor-type radiation defects being formed. As a result, the concentration difference N_d_–N_a_ (N_a_–N_d_) linearly decreases with increasing irradiation dose.
- It was shown that the dose *Φ*_cr_ corresponding to the total degradation of a device satisfies the condition *Φ*_cr_ ≈ V_d_/n_0_, where V_d_ is the removal rate of electrons from the blocking layer of the device and n_0_ the initial electron concentration in the blocking (drift) layer. In the VMOSFET devises under study (1.2 kV class), *Φ*_cr_ ≈ 10^14^ cm^−2^, from a physics viewpoint, the condition *Φ*_cr_ ≈ V_d_/n_0_ reflects the situation in which the concentration of a deep levels created by irradiation becomes equal to the initial concentration of electrons in the drift region.
- The radiation hardness of SiC devices exceeds by approximately two orders of magnitude that of silicon-based devices with the same breakdown voltage.
- It was shown for the first time that when operating under conditions of increased radiation and elevated temperature, the service life of carbide devices is longer than that for the same devices working at room temperature. Judging by the carrier removal rate or the increase in the base resistance of Schottky diodes under irradiation, it can be said that the service life is at least doubled.

## Figures and Tables

**Table 1:** Comparison of carrier removal rates in devices based on SiC and Si.

SiC Device Type	SBD 600 V	SBD 1200 V	JBS 1700 V	Si
N_d_–N_a_ in base, cm^−3^	6.6 × 10^15^	4.5 × 10^15^	3.5 × 10^15^	~10^15^
V_d_ for electrons (0.9 MeV), cm^−1^	0.095 [32]	0.073 [32]	0.12 [33]	0.232 [34]
V_d_ for protons (15 MeV) cm^−1^	63 [33]	50 [33]	54 [33]	110 [35]

**Table 2:** Annealing temperatures of radiation defects in SiC after various kinds of irradiation [38].

Refs.	SiC	Kind of Irradiation	Onset Temperature of the Defect Rearrangement, °C	Temperature at Which the RDs are Finally Annealed-Out, °C
[40]	4H-p	e-2.5 MeV	200–400	950–1400
[41]	6H-n	e-0.3–0.4 MeV	400–900	1600
[42]	4H-n	e-15 MeV; p-1.2 MeV	200–800	>1200
[43]	6H-n 4H-n	e-2.5 MeV; p-1 MeV, He	-	1200–1700
[44]	4H-n	e-15 MeV	400–800	1200–2000
[45]	6H,4H,15R-p	p-8 MeV	-	>1200

e stands for irradiation with electrons; p, for irradiation with protons; and He, for irradiation with helium nuclei.

## Data Availability

The data underlying this article will be shared on reasonable request from the corresponding author.
